# Correlates of obsessive-compulsive and related disorders symptom severity during the COVID-19 pandemic

**DOI:** 10.1016/j.jpsychires.2021.03.046

**Published:** 2021-04-13

**Authors:** Leonardo F Fontenelle, Lucy Albertella, Mary-Ellen Brierley, Emma M Thompson, Louise Destrée, Sam R Chamberlain, Murat Yücel

**Affiliations:** 1 Turner Institute for Brain and Mental Health, Monash University, 770 Blackburn Road, Clayton, Victoria 3168, Australia; 2 Obsessive, Compulsive, and Anxiety Spectrum Research Program. Institute of Psychiatry, Federal University of Rio de Janeiro (UFRJ) & D’Or Institute for Research and Education (IDOR), Rio de Janeiro, Brazil; 3 Department of Psychiatry, Faculty of Medicine, University of Southampton, & Southern Health NHS Foundation Trust, Southampton, UK

## Introduction

Obsessive-compulsive and related disorders (OCRDs) comprise a recently recognized group of disorders sharing repetitive thoughts and/or behaviors, key diagnostic validators, and underlying etiology. They include obsessive-compulsive disorder (OCD), body dysmorphic disorder (BDD), hoarding disorder (HD), trichotillomania (TTM; hair pulling disorder), and excoriation (skin picking) disorder (SPD) in the DSM-5. Although the precise prevalence of OCRDs varies depending on the study and context (Buhlmann et al., 2010; Grant et al., 2020; Hayes et al., 2009; Postlethwaite et al., 2019; Ruscio et al., 2010), it is possible to estimate that a substantial proportion of the world population exhibit at least one current OCRD, and an even larger group of individuals experience subthreshold symptoms.

Evidence suggests OCRDs are associated with increased disability, costs and mortality. For instance, OCD, the paradigmatic OCRD, has been described as the 6th leading psychiatric disorder in terms of Disability Adjusted Life Years (DALYs) (Hollander et al., 2016). Different studies have now shown a decrease in several quality of life domains, as well as increased suicidality, in people with a range of OCRDs, including OCD (Angelakis et al., 2015; Coluccia et al., 2016), BDD (Angelakis et al., 2016; IsHak et al., 2012), and HD (Chakraborty et al., 2012; Tolin et al., 2019). More recently, evidence has also emerged linking OCD (Isomura et al., 2018) and HD (Darke and Duflou, 2017; Tolin et al., 2008) to an increased risk of metabolic and cardiovascular complications, which further increase morbidity and early mortality (Meier et al., 2016). Thus, it is imperative to identify the risk factors for OCRDs to minimize the burden caused by this group of illnesses.

Among risk factors for OCRDs, and mental illness more broadly, are stressful life or traumatic events (SLE). Meta-analytic studies suggest that these events can have a major role in precipitating OCD in predisposed individuals (Miller and Brock, 2017). Likewise, evidence is starting to emerge from cross-sectional studies linking other OCRD [such as BDD (Didie et al., 2006;Semiz et al., 2008; Valderrama et al., 2020) and HD (Cromer et al., 2007; Landau et al., 2011; Tolin et al., 2010b)] to similar SLEs. Yet, the nature (or “content”) of SLEs more likely to precipitate OCRDs is unclear. It is still not known for instance, if certain events may be particularly likely to give rise to specific OCRD phenotypes. Some preliminary connections have been found: for example, a case series described that exposure to blood and human tissue was related to a recurring feeling of contamination and to washing rituals (Sasson et al., 2005).

In 2020, the world has witnessed an unprecedented pandemic that has affected humanity in many different ways (Sanderson et al., 2020). Firstly, COVID-19 has posed a severe threat to people’s health for being highly contagious with tremendous death tolls. Secondly, the response of most countries, which has included severe lockdown and social distancing measures, has lead to increased social isolation and decreased participation in meaningful activities, with significant implications for the mental health of their citizens. Finally, the economic consequences of the COVID-19 have proved to be far reaching, as the resulting job losses and financial insecurity can clearly be detrimental to one’s mental health and overall quality of life (Dawel et al., 2020).

For the reasons listed above, it has been speculated that the impact of the COVID-19 pandemic on OCD and HD would be colossal (Banerjee, 2020; Fontenelle and Miguel, 2020). Yet, results have been mixed, with some studies reporting deterioration of symptoms (Benatti et al., 2020; Davide et al., 2020; Jelinek et al., 2020; Littman et al., 2020; Matsunaga et al., 2020; Nissen et al., 2020; Tanir et al., 2020), some describing no change (Benatti et al., 2020; Chakraborty and Karmakar, 2020; Littman et al., 2020), and others reporting even improved symptoms (Kuckertz et al., 2020; Littman et al., 2020; Perkes et al., 2020). As these outcomes are likely to reflect individual differences, different types of and levels of exposure to SLEs related to the COVID-19, and different OCD phenotypes, the present study was devised. In this online study, we had two main objectives: 1) to retrospectively evaluate whether general symptoms of OCRDs in the general population (i.e. OCD, BDD, HD, TTM and SPD) have worsened due to COVID-19 pandemic and whether that worsening translated into increased prevalence of clinically significant rates; and 2) to investigate which demographic or clinical factors were related to the worsening of specific OCRDs.

We predicted that OCD and HD would worsen due to the pandemic (Banerjee, 2020; Fontenelle and Miguel, 2020). More specifically, we did hypothesize, based on prior more general literature, that particular characteristics of OCD and HD would be linked to greater untoward impact of the pandemic (e.g. female gender, lower levels of education, people from racial minorities, non-married subjects, unemployed participants, and those with greater personal and family histories of psychopathology) (Brewin et al., 2000). We also hypothesized that greater compulsivity traits (Albertella et al., 2020) and preexisting contamination OCD symptoms (Abba-Aji et al., 2020; Davide et al., 2020; Fontenelle and Miguel, 2020; Matsunaga et al., 2020; Tanir et al., 2020) would predict worse post-COVID-19 OCD symptoms; that more impulsivity traits (Timpano et al., 2013; Timpano and Schmidt, 2013) would predict greater hoarding after the pandemic; and that schizotypal traits (Volz and Heyman, 2007) would predict increased “mental contamination” beliefs (Rachman, 2006) [i.e. “a sense of internal dirtiness” elicited by *intangible* stimuli, such as unwanted or repulsive thoughts or images”(Blakey and Jacoby, 2018)] during the stress of the pandemic. We didn’t have specific predictions but explored whether the remaining OCRDs (BDD, TTM and SPD) were affected by the pandemic.

## Methods

### Participants

Adult participants (≥ 18 years of age) were recruited for this cross-sectional study through Amazon Mechanical Turk (AMT). The advertisement for the study was made available to all workers on the platform who resided in the United States, were over the age of 18, and had English as their first language or learnt English before the age of 7 (as all questionnaires were in English). Once participants consented to taking the survey, interested participants were directed to a Qualtrics-based series of questionnaires (see below), where informed consent was given.

The AMT is an American online crowdsourcing platform in which workers can browse Human Intelligence Tasks by keyword, compensation, availability, and qualifications (McKay et al., 2018). Shapiro et al (Shapiro et al., 2013) demonstrated that the prevalence of mental health problems identified in AMT studies were similar or higher than in the general population. In their specific study (Shapiro et al., 2013), the AMT assessments were considered valid by being associated with established demographic predictors (unemployment) and also displayed adequate internal and test-retest reliability. Importantly, participants of the Shapiro et al. study felt particularly confortable disclosing mental health information online.

Our survey took approximately 90 minutes to complete, after which time participants received a code to be entered in Mechanical Turk website to be reimbursed US$15. Participants could leave the survey and come back within 24 hours to complete it. Yet, to maximize the validity of the survey results, individuals could not attempt the survey twice. All study procedures were carried out in accordance with the Declaration of Helsinki, and participants provided informed consent. The Monash University Human Research Ethics Committee ethically reviewed and approved the study.

### Assessment

#### Demographics

Participants responded to a questionnaire that included information on age, gender, education (less or higher than college), ethnicity (white vs. non-white), marital status (married vs. non married), and employment status (employed vs. non-employed). Patients were also asked about whether they had received a previous diagnosis of any OCRD by a health practitioner and whether they had any family history of OCD, BDD, HD, TTM or SPD symptoms.

#### Coronavirus related stress

The Coronavirus Traumatic and Stressful Life Events Scale (COROTRAS) is a self-report inventory that lists 16 potential life events related to the COVID-19 pandemic (e.g. “have you lost your job or had a reduction in your salary as a consequence of the COVID-19 pandemic?”) (Fontenelle et al., 2020a). Through the COROTRAS, the respondent can indicate whether he or she has experienced these events, whether they found the event stressful, and rate the intensity of a spectrum of emotions (fear, helplessness, anger, sadness, guilt, shame and disgust) that he or she might have experienced as a consequence of the exposure to their most stressful event related to the coronavirus pandemic.

The COROTRAS generates (1) the total number of life changes related to coronavirus, (2) the total number of SLE related to coronavirus and (3) the intensity of each emotion experienced as a result of the most stressful coronavirus event, ranging from 0 (absent) to 4 (extreme). Intraclass correlation coefficient of the COROTRAS was considered excellent (Cronbach’s alpha = .917) (Fontenelle et al., 2020a). Prior inspection of the correlations between the COROTRAS subscores and DASS 21 revealed the scale to have acceptable convergent validity (Fontenelle et al., submitted). For the purposes of this study, we used the total number of SLE related to coronavirus.

#### Severity of OCRD symptoms and other quantitative measures

Questions from each OCRD measure were adapted so that subjects would answer how they were feeling currently (i.e. during the pandemic) and before the COVID-19 pandemic. Contextually, participants completed the survey between July 29^th^ and July 30^th^, which corresponded to a time when major changes in the lifestyle (such as lockdowns, social distancing and high rates of COVID-19 transmission) were taking place.

##### Obsessive-Compulsive Symptoms

The Dimensional Obsessive-Compulsive Scale (DOCS) is a 20-item self-report questionnaire that evaluates the severity of four dimensions of OCD symptoms that have been most reliably replicated in different studies, including contamination, fear of harm, unacceptable thoughts, and symmetry. For each symptom dimension, five different features (time spent, avoidance, distress, interference and control) are assessed and measured on a scale from 0 to 4 (Abramowitz et al., 2010). Subscale scores are obtained by summing the five items of each subscale (range = 0-20), which are summed to obtain total score (range = 0-80) (Abramowitz et al., 2010). The DOCS has demonstrated excellent psychometric characteristics. The DOCS’s cut-off score is 21.

##### Mental Contamination

The Vancouver Obsessional Compulsive Inventory – Mental Contamination (VOCI-MC) is a 20-item self-report instrument that quantifies the severity of mental contamination symptoms. Respondents are asked how much they agree with twenty statements about mental contamination symptoms (e.g. “I often feel dirty under my skin”, “I often feel dirty or contaminated even though I haven’t touched anything dirty”; or “I often feel the need to cleanse my mind”). Answers vary from 0 (Not at all) to 4 (Very much) for each item, leading to a maximum overall scale of 80 (Radomsky et al., 2014). The VOCI-MC has demonstrated adequate psychometric properties.

##### Body Dysmorphic Symptoms

The Appearance Anxiety Inventory (AAI) is a 10-item self-report tool to quantify the severity of the responses to a distorted body image, particularly avoidance behavior and threat monitoring (e.g. “I compare aspects of my appearance to others”) (Veale et al., 2014). Participants are asked to select the response that best describes the way they felt about the appearance of a specific feature over the past week, with responses to each item ranging from 0 (not at all) to 4 (all the time) (Veale et al., 2014). The total score is the sum of all responses. The AAI has demonstrated appropriate psychometric characteristics (Veale et al., 2014). The AAI’s cut-off score for BDD is 19.

##### Hoarding Symptoms

The Hoarding Rating Scale-Self Report (HRS-SR) is a six-item instrument based on the original interview (Tolin et al., 2010a; Tolin et al., 2008). The HRS-SR evaluates severity of clutter, difficulty discarding, excessive acquisition, distress, and impairment (Tolin et al., 2008). Each item (structured as questions) can generate of scores ranging from 0 (none) to 8 (extreme). Total scores include the summation of all responses. The HRS-SF has demonstrated adequate psychometrics properties (Tolin et al., 2008). Sensitivity and specificity analyses indicate that the HRS has a total clinical cutoff score of 14 (Tolin et al., 2010a).

##### Hair pulling

The Massachusetts General Hospital Hairpulling Scale (MGHHS; (Keuthen et al., 1995) is a seven-item self-report instrument that quantifies the severity of hair pulling in the previous week by assessing urges to pull hair, time spent pulling, perceived control, and distress associated with pulling. In the MGHHS, each item is scored on a 5-point scale from 0 (no symptoms) to 4 (severe symptoms). Items scores are summed to produce a total score (range 0 to 28). The MGHHS has shown acceptable psychometric features (Keuthen et al., 1995). A cut-off score of 17 for clinical significance has been suggested (Solley and Turner, 2018).

##### Skin Picking

The Skin Picking Scale-Revised (SPS-R; (Snorrason et al., 2012)) is an eight-item self-report instrument that quantified the severity of skin picking in the previous week by assessing urges to pick skin (frequency/intensity), time spent, control, distress, interference, avoidance, and damage associated with skin picking. In the SPS-Revised, each item is scored on a 5-point scale from 0 (no symptoms) to 4 (severe symptoms). Items scores are summed to produce a total score (range 0 to 24). The SPS has shown acceptable psychometric features (Snorrason et al., 2012). A cut-off score of 9 for clinical significance has been suggested (Solley and Turner, 2018).

##### Psychological Distress

The Depression Anxiety Stress Scale - 21 (DASS-21; (Lovibond and Lovibond, 1995)) is a 21-item self-report questionnaire, based on the original 42 item scale, that quantifies negative affective experiences in the past week. In the DASS – 21, respondents are asked to rate how much a specific statement applies to them using a 4-point Likert scale that varies from 0 (‘did not apply to me at all’) to 3 (‘applied to me very much’). The DASS-21 generates three different subscores, namely, depression, anxiety, and stress reactivity. A total score is obtained by summing all subscales. The DASS-21 has shown excellent psychometric properties in a variety of contexts. (Sinclair et al., 2011).

##### Disability

The 12-item World Health Organization Disability Assessment Schedule 2.0 (WHODAS 2.0) is a self-report instrument that quantifies functional impairments in the past thirty days (Andrews et al., 2009). Participants are presented with 12 statements describing different daily activities (e.g., “Taking care of your household responsibilities”) and asked whether they have any difficulty performing them. Responses range from 0 (none) to 4 (extreme or cannot do) The WHODAS 2.0 has shown excellent psychometric characteristics in non-clinical (Andrews et al., 2009) and clinical (Axelsson et al., 2017) settings. Total scores are obtained from summing up responses to each item (ranging from 0-48). Greater scores reflect greater disability.

##### Quality of life

The short-form version of the Quality of Life, Enjoyment, and Satisfaction Questionnaire-Short Form (Q-LES-Q-SF) is a 16-item self report instrument that assess satisfaction or enjoyment related to physical health, medications, feelings, work/school, household duties, leisure-time activities, social relations, and general activities (Endicott et al., 1993). A 4-point Likert scale ranging from 1 (very poor) to 5 (very good) follows each question. Responses to the questions are summed up to generate total scores between 14 and 70. Greater scores reflect poorer enjoyment and satisfaction. The Q-LES-Q-SF has shown appropriate psychometric properties. (Stevanovic, 2011).

##### Compulsivity-Impulsivity Traits

Compulsivity and impulsivity traits, thought to be particularly relevant for OCRDs, were assessed with the Cambridge-Chicago Compulsivity Trait Scale (CHIT) (Chamberlain and Grant, 2018) and the Barratt Impulsivity Scale (BIS) (Stanford et al., 2009). The CHIT (Chamberlain and Grant, 2018) is a 15-item scale covering the need for completion or perfection, being stuck in a habit, reward-seeking, desire for high standards, and avoidance of situations that are hard to control. Each item is scores from 0 (“strongly disagree”) to 3 (“strongly agree”). The BIS-11 (Stanford et al., 2009) is a 30 item scale that measures the individual tendency to think and behave impulsively. The subject must assess whether each item applies to him/her and rate them according to a Likert scale raging from 1 (rarely or never) to 4 (almost always/always). Total scores of the CHIT and the BIS were used.

### Statistical analyses

Descriptive statistics were described in percentages; means and standard deviations (for normal distributions) or medians and range (minimum-maximum) (for non-normal distribution). Quantitative variables (i.e. DOCS and other scales measuring symptom severity) were compared between two time points (pre vs. during COVID-19) using Wilcoxon Signed Ranks tests. Qualitative variables (i.e. rates of people showing persistent, absent, *de novo*, and remitting OCRDs) were compared using McNemar tests. For each OCRD symptom that worsened during COVID-19, we also planned to perform regression analyses that considered as the dependent variable the severity of the current (COVID-19) OCRD symptom.

A negative binomial regression was chosen based on the distribution of the data, which was skewed. Independent variables included the severity of the specific pre-COVID-19 OCRD symptoms being regressed and a number of independent variables hypothesized to be related to greater chance of symptoms’ deterioration, such as sociodemographic factors, the number of COVID-19 related events (stressful or not), severity of compulsivity/impulsivity symptoms, intensity of schizotypal traits, and severity of affective (depression, anxiety and stress) symptoms. The level of statistical significance was set at .05.

## Results

### Descriptive statistics

The sample included 829 subjects (52.6% females). They declared being from the US in 98.5% of cases (in 1.5%, the information regarding origin was missing). Mean age at assessment was 38.52 (SD 12.69) years (minimum 18 and maximum 82 years). The majority of the sample was white (72.2%), had at least college education (91.1%), and was employed (45%). Subjects reported being married in 45% of the cases. Most subjects (55.7% of the sample) declared not having a history of a previous mental illness diagnosis, and in 54.2% of the cases no family history of mental illness was reported.

### OCRDs symptoms before vs. during COVID-19

**Figure 1:**
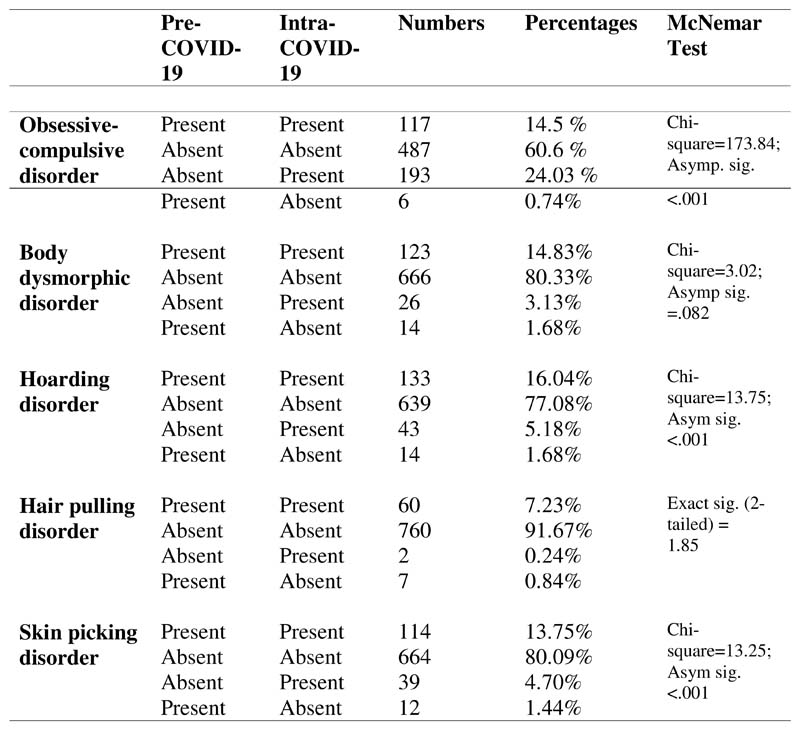
Number of subjects exhibiting persistent, absent, *de novo*, and remitting OCRDs across the COVID-19 pandemic

As data was not normally distributed, for each construct, medians (and minimum and maximum values) are described for the two time points (pre vs. during COVID-19; table 1). As can be seen in table 1, scores for all OCRDs (with the exception of BDD) increased significantly after the pandemic. There were also significant increases in disability levels and depression, anxiety and stress scales, along with significant decreases in quality of life, enjoyment and satisfaction. The frequency of individuals displaying clinically significant OCRD symptoms (according to published cut-off scores for each scale) are depicted and contrasted before and during COVID-19 in table 1. Significantly increased rates were observed for OCD, HD and SPD. Figure 1 describes the numbers of people that exhibited persistent, absent, *de novo*, or remitting OCRDs.

### Predictors of severity of COVID-19 OCRD symptoms

For each OCRD symptom that worsened during COVID-19 (OCD, HD and SPD), we performed regression analyses that considered the severity of the current (intra-COVID-19) OCRD symptom as the dependent variable and a number of independent variables hypothesized to be related to greater chance of symptoms’ deterioration such as sociodemographic factors (i.e. age, gender, educational levels, marital status ethnicity, and employment status), personal history and family history of the specific OCRD diagnosis, COVID-19 related events (stressful or not), compulsivity/impulsivity levels, schizotypal symptoms, depression, anxiety and stress levels, and the pre-covid 19 severity of the specific OCRD symptom under investigation (tables 2 to 4). Inspection of the histogram of scores in different OCRD scales revealed a skewed distribution, leading us to choose a negative binomial regression. All VIF levels were within acceptable limits. Similar models performed for BDD and TTM symptoms are included in the appendix.

As seen in table 2, increased DOCS scores during COVID-19 were predicted by female gender (*B* = -.167, *SE* = .077, *p* = .031), a higher number of stressful events related to the COVID-19 pandemic (*B* = .056, *SE* = .024, *p* = .018), higher compulsivity levels (*B* = .026, *SE* = .0068, *p* < .001), and higher pre-COVID-19 DOCS scores (*B* = .038, *SE* = .0048, *p* < .001). In contrast, increased scores in the HRS after COVID-19 were predicted by lack of a diagnosis of HD by a clinician (*B* = 2.708, *SE* = 1.052, *p* = .010), higher compulsivity levels (*B* = .019, *SE* = .0074, *p* = .011), increased severity of schizotypal traits (*B* = .023, *SE* = .0099, *p* = .019), and increased severity of hoarding symptoms before the pandemic (*B* = .160, *SE* = .0086, *p* < .001). Finally, increased severity of pre-existing skin picking was the only predictor of severity of skin picking during the COVID-19 (*B* = .065, *SE* = .0170, *p* < .001).

Two additional regression models were performed for OCD symptoms. The first one also included COVID-19 DOCS scores as the dependent variable, but this time with specific pre-covid DOCS subscores (fear of harm, contamination, symmetry and unacceptable thoughts) controlling for the same sociodemographic factors described previously and also for depression, anxiety, and distress (table 5). The second one addressed post-COVID-19 VOCI-MC scores as the dependent variable along with sociodemographic information, personal history and family history of a diagnosis of OCD, COVID-19 related events (stressful or not), compulsivity/impulsivity levels, schizotypal symptoms, depression, anxiety and stress levels, and the pre-covid-19 severity of mental contamination symptoms (table 6).

As in the model listed in table 2, female gender (*B* = -.153, *SE* = .0764, *p* < .045), more stressful events related to the COVID-19 and more compulsivity levels (*B* = .064, *SE* = .0233, *p* = .006) emerged as significant predictors of the severity of post-COVID-19 obsessive-compulsive symptoms in this different model (see table 5). However, pre-covid “fear of harm” (*B* = .069, *SE* = .0176, *p* < .001), and “symmetry” (*B* = .052, *SE* = .0175, *p* = .003) also predicted post-COVID-19 DOCS scores (table 5). Finally, mental contamination was predicted by non-white ethnicity (*B* = .208, *SE* = .0900, *p* = .021), number of stressful events related to the COVID-19 (*B* = .056, *SE* = .0257, *p* = .028), compulsivity levels (*B* = .035, *SE* = .0070, *p* <.001), severity of schizotypal traits (*B* = .031, *SE* = .0093, *p* = .001), and pre-covid mental contamination symptoms (*B* = .069, *SE* = .0056, *p* < .001).

## Discussion

In this cross-sectional online study, we investigated self-reported symptoms of different OCRDs (namely OCD, BDD, HD, TTM and SPD) before and during the COVID-19 pandemic in a sample of 829 subjects (largely from the USA) selected through Amazon Mechanical Turk at the end of July 2020. Our main findings can be summarized as the following: Firstly, OCD, HD, TTM and SPD symptoms significantly worsened after the pandemic, along with increased disability, more affective (anxiety, depressive, and stress) symptoms and declined quality of life. However, no significant difference between pre- and intra-covid rates of *clinically significant* BDD and TTM symptoms were noted. Secondly, female gender, the number of COVID-19 related stressful events, and pre-COVID-19 fear of harm and symmetry symptoms predicted OCD symptoms during the pandemic. Thirdly, lack of a HD diagnosis by a mental health professional and worse severity of schizotypal symptoms predicted current hoarding symptoms. Lastly, compulsivity traits predicted more severe OCD and HD symptoms during the COVID-19 pandemic.

The fact that a substantial proportion of people reported developing clinically significant OCD and HD symptoms during the COVID-19 pandemic is consistent with early theoretical speculations (Banerjee, 2020; Fontenelle and Miguel, 2020) and empirical findings suggesting that COVID-19 represents a threat to individuals showing predisposition towards these symptoms (Benatti et al., 2020; Matsunaga et al., 2020; Nissen et al., 2020). On the other hand, the reason why BDD symptoms did not deteriorate may be partly attributable to the lockdown measures, which might have decreased the distress associated with going out with what participants believe to be an appearance problem (Pikoos et al., 2020). Finally, while both TTM and SPD symptoms were reported to have worsened, prevalence of clinically significant TTM did not increase after the pandemic. It is difficult to explain these differences, as both TTM and SPD are very similar from the sociodemographic and clinical point of view (Lochner et al., 2002). However, whereas SPD symptoms might be more likely to be triggered by the individuals’ sight in the mirror (which can be considered more likely to occur during lockdown) (Odlaug and Grant, 2008), TTM symptoms-associated distress may diminish as a consequence of decreased social exposure in TTM individuals prone to greater to social anxiety (Lochner et al., 2002).

Notably, female gender, the number of COVID-19 related stressful events, compulsivity levels, and, in a separate model, pre-COVID-19 fear of harm and symmetry symptoms, predicted OCD symptoms during the pandemic. Our findings support previous studies showing relatively greater vulnerability of adult women to stress (Hodes and Epperson, 2019) and the usefulness of our scale to assess the totality of COVID-19 stressful events. Nevertheless, in contrast with our initial hypothesis, previous severity of contamination and washing did not emerge as predictors of “intra-covid” severity of OCD. Perhaps as a consequence of prolonged lockdown measures, OCD symptoms that tend to occur at home, such as symmetry and fear of harm, were more likely to determine OCD deterioration. They may represent, for instance, compulsions to rearrange personal belongings at subjects’ own residences, aggressive impulses towards family members (Moreira and Pinto da Costa, 2020), or the fear for the lives of relatives falling sick or dying (Nissen et al., 2020). It is also possible that current contamination and washing symptoms were less likely to be reported for being now validated by society in general (Perkes et al., 2020).

Mental contamination, defined as an internal feeling of dirtiness experienced in the absence of contact with a physical contaminant (Rachman, 1994), was predicted by non-white ethnicity, number of COVID-19 stressful events, compulsivity levels, and schizotypal symptoms. These findings may be indicative of the potential influence of cultural background on the nature of OCD symptoms experienced; e.g. a pattern of culturally related beliefs (Subbotsky and Quinteros, 2002) that may be relevant to contamination concerns (Speltini and Passini, 2014) and related to magical thinking (Tolin et al., 2001) (or sympathetic magic (Tolin et al., 2004)). While compulsivity and COVID-19-related SLEs as shared risk factors do approximate mental contamination and typical OCD, our findings also suggest people high on schizotypal traits (who tend to hold delusional like-ideas) may be more likely to display magical thinking (Eckblad and Chapman, 1983) that includes atypical forms of contamination.

Consumer panic or stockpiling for the fear of running out of essential goods might have led to a significant reported worsening of HD to clinically significant levels or appearance of *de novo* HD cases (Banerjee, 2020; Dammeyer, 2020; Keane and Neal, 2020; Micalizzi et al., 2020; Oosterhoff and Palmer, 2020). Accordingly, a model that included the lack of a previous HD diagnosis by a mental health professional, higher compulsivity levels, and severity of schizotypal symptoms statistically predicted hoarding symptoms during the COVID-19 pandemic. Thus, it is likely help-seeking behavior (including some sort of treatment being delivered, therapeutic support or even greater insight about the subjects’ own behavior) protected individuals from showing HD symptom deterioration during the COVID-19 pandemic. Accordingly, previous studies have already demonstrated a close relationship between hoarding and schizotypal traits, both in clinical and non-clinical (Weintraub et al., 2018) samples. We now demonstrated that schizotypal traits might engender vulnerability for hoarding symptoms, particularly in relation to the COVID-19 pandemic. These findings are also consistent with schizotypal traits conferring greater vulnerability to stress (Grattan and Linscott, 2019; Walter et al., 2018).

“Compulsivity” traits conferred greater self-reported susceptibility to a range of mental health problems, including more severe COVID-19 pandemic obsessive-compulsive, mental contamination, and hoarding symptoms. These findings are consistent with compulsivity traits having major transdiagnostic implications, (Chamberlain and Grant, 2018; Figee et al., 2016; Fontenelle et al., 2020b) as initially reported in the study by Albertella et al. (Albertella et al., 2021). They may be particularly relevant in the presence of major stressful events with “contents” that, by matching underlying vulnerabilities, are able to contribute to deterioration in OCD/HD symptoms and lead to conversion from subclinical or no symptom to clinically relevant symptoms. These events, including the threats posed by COVID-19 infection and the social distancing enforced by different international health agencies, may explain why OCD and HD sharing higher compulsivity levels may be more closely related to each other and likely to deteriorate *pari passu*. One study suggested that financial problems (and the threats of deprivation) might impact negatively the response of OCD patients to exposure and response prevention. (Storch et al., 2021)

This study has a number of limitations. Firstly, it was an online survey and was not designed to be epidemiologically representative of a particular population. Like other Amazon Mechanical Turk samples, it included a relatively high proportion of white, highly educated females (Moss et al., 2020). Thus, the high numbers described here, particularly those related to d*e novo* cases, may not fully generalize to the population at large. They do, however, represent rates that need to be considered in studies performed in other (e.g. epidemiological) contexts. Secondly, our study included “before” approximations of symptoms severity in relation to the COVID-19 pandemic measured cross-sectionally. As these assessments relied on patients’ memory, they may be subject to a number of biases. The validity of retrospective assessments is likely to be lower than longitudinal data collection, but the unexpected nature of the pandemic means that such longitudinal studies with appropriate ‘baseline’ data are scarce. For this reason, we acknowledge that follow up assessments would be ideal to assess the significance of our findings, which may prove relevant in future waves of the pandemic.

Yet, we feel that at least two factors contribute to minimize recall bias in the present study. The unparalleled magnitude and severity of the pandemic may have provided a clear differentiation between participants’ mental state before and after the onset of the health crisis. Also, the temporal proximity of the present assessment to the onset of the COVID-19 pandemic may have facilitated a more accurate recall by the participants. Finally, another potential limitation of our study is geographic and time coverage, as data collection was restricted to the US in late July 2021, thus limiting generalizability. Nevertheless, one could also argue that US cities were under different infection rates, lockdown policies, and adherence to social distance practices. Clearly, in the context of a pandemic such as the COVID-19, it may be difficult to balance sample homogeneity vs. representativeness.

Our study has several implications for clinical practice. It suggests clinicians must be aware that community individuals may deteriorate and be exposed to significantly higher rates of *de novo* cases of a range of OCRDs (and not only OCD). Further, although there were initial concerns in the literature about the role of contamination concerns as predicting symptom aggravation during the pandemic [and how best to manage these symptoms clinically (Fineberg et al., 2020; McKay et al., 2020; Sheu et al., 2020)], our data suggest that other symptom dimensions (fear of harm and symmetry) are important determinants of OCD worsening. This finding raises concerns about how to treat these individuals in the presence of strict lock down measures. Nevertheless, exposure to increased threat levels as a consequence of the pandemic and greater time spent at home may also provide great opportunities for exposure and response prevention. Accordingly, the development of online therapies for OCRDs in different cultures should be pursued.

The current findings suggest that a diagnosis of clinically significant HD by clinical teams may increase awareness and insight and also ease symptom deterioration related to the pandemic. Alternatively, lack of a formal diagnosis may reflect less treatment seeking, less insight, and more vulnerability to SLEs. Further, although the evidence supporting specific treatments for people with high schizotypal traits is sparse, atypical antipsychotics (such as risperidone and olanzapine) appear to be helpful in some cases (Kirchner et al., 2018). Potentially, in specific cases, treatment of schizotypal traits may help to decrease hoarding and alleviate mental contamination symptoms. Although preliminary evidence supports the use of serotonin reuptake inhibitors in people with obsessive-compulsive personality features (a construct that partially overlaps with compulsivity) (Ansseau et al., 1991; Ekselius and von Knorring, 1998), there is also current interest in the efficacy lifestyle interventions that may be able to redirect patients compulsivity traits toward healthier behaviors (Fontenelle et al., 2018).

In conclusion, this study indicates that the unprecedented distress that resulted from the COVID-19 pandemic in 2020 includes significant aggravation of several OCRD symptoms in the general population, particularly of OCD, HD and SPD. Increased vulnerability to symptom worsening may relate to specific sociodemographic and clinical characteristics, including gender, previous diagnosis and treatment seeking, specific OCD symptoms, and severity of compulsivity and schizotypal traits, and the amount of stress people experienced related to the COVID-19 pandemic. This information may prove valuable for preventative initiatives in relation to this and future waves of pandemics.

## Supplementary Material

Appendix

## Figures and Tables

**Table 1:** Clinical characteristics before vs. during the COVID-19 pandemic

	Before COVID-19		During COVID-19		Wilcoxon Signed Ranks
					
**Severity of symptoms**	Medians (min-max)		Medians (min-max)		
DOCS	6 (0-65)		16 (0-74)		Z=-20.857; p<.001
VOCI-MC	4 (0-66)		7 (0-80)		Z=-15.424; p<.001
AAI	6 (0-40)		6 (0-40)		Z=-1.553; p<.120
HRS	3 (0-35)		3 (0-37)		Z=-4.364; p<.001
MGH-HPS	0 (0-28)		0 (0-22)		Z=-4.579; p<.001
SPD	0 (0-32)		0 (0-32)		Z=-4.587; p<.001
DASS	6 (0-55)		10 (0-57)		Z-13.701; p<.001
					
**Disability levels**					
WHODAS	15 (12-49)		17 (12-56)		Z=-14.031; p<.001
					
**Quality of life**					
Q-LES-Q-SF	54 (19-70)		50.00 (19-70)		Z=-15.042; p<.001
					
**Rates of OCRDs**	Percentages		Percentages		**McNemar Test**
OCD (DOCS ≥ 21)	15.3%		38.6%		Chi-square=173.84; Asymp. sig. <.001
BDD (AAI ≥19)	16.5%		18.0%		Chi-square=3.02; Asymp sig. =.082
HD (HRS ≥ 14)	17.7%		21.2%		Chi-square=13.75; Asym sig. <.001
TTM (MGH-HPS ≥ 17)	8.1%		7.5%		Exact sig. (2-tailed) = 1.85
SPD (SPS ≥ 9)	15.2%		18.5%		Chi-square=13.25; Asym sig. <.001

Footnote: DOCS=Dimensional Obsessive-Compulsive Scale; VOCI-MC=Vancouver Obsessional Compulsive Inventory-Mental Contamination Scale; AAI=Anxiety Appearance Inventory; HRS=Hoarding Rating Scale; MGH-HPS=Massachusetts General Hospital Hair Pulling Scale; SPRS=Skin Picking Scale; WHODAS= World Health Organization Disability Assessment Schedule; Q-LES-Q-SF=Quality of Life Enjoyment and Satisfaction Questionnaire Short Form; DASS-21= Depression Anxiety Stress Scale-21; OCD=Obsessive-Compulsive Disorder; BDD=Body Dysmorphic Disorder; HD=Hoarding Disorder; TTM=Hair Pulling Disorder; SPD=Skin Picking Disorder;

**Table 2:** Negative binomial model with intra COVID-19 pandemic scores on the Dimensional Obsessive-Compulsive Scale

Parameter Estimates									
Parameter	B	Std. Error	95% Wald Confidence Interval		Hypothesis Test			Collinearity Statistics	
			Lower	Upper	Wald Chi-Square	df	Sig.	Tolerance	VIF
(Intercept)	1.536	.4108	.731	2.342	13.986	1	.000		
Age	-.003	.0033	-.009	.004	.733	1	.392	.847	1.180
**Male (vs. other) gender**	**-.167**	**.0776**	**-.319**	**-.015**	**4.648**	**1**	**.031**	.944	1.060
Lower (vs. higher) education levels	-.129	.1349	-.394	.135	.918	1	.338	.951	1.052
Non-white (vs. white) ethnicity	.099	.0872	-.072	.270	1.287	1	.257	.922	1.084
Non-married (vs. married) status	-.057	.0804	-.215	.100	.507	1	.477	.897	1.115
Unemployed (vs. employed)	.008	.1561	-.298	.314	.003	1	.957	.970	1.031
Lack vs. presence of past OCD diagnosis	.197	.2056	-.206	.600	.916	1	.338	.860	1.163
Negative (vs. positive) family history of OCD	.017	.1592	-.295	.330	.012	1	.913	.900	1.111
Number of COVID-19 related events	.033	.0316	-.029	.094	1.062	1	.303	.488	2.048
**Number of COVID-19 related *stressful* events**	**.056**	**.0238**	**.010**	**.103**	**5.641**	**1**	**.018**	.499	2.006
**CHIT total**	**.026**	**.0068**	**.013**	**.040**	**14.985**	**1**	**<.001**	.702	1.425
BIS total	-.001	.0087	-.018	.016	.010	1	.922	.816	1.225
SPQ total	.017	.0090	.000	.035	3.749	1	.053	.628	1.593
DASS21 depression (before)	.001	.0126	-.024	.026	.005	1	.944	.392	2.549
DASS21_anxiety (before)	.006	.0202	-.034	.045	.079	1	.778	.406	2.462
DASS21_stress_(before)	-.010	.0167	-.043	.023	.352	1	.553	.327	3.059
**DOCS_total_(before)**	**.038**	**.0048**	**.028**	**.047**	**62.357**	**1**	**<.001**	.555	1.803
(Scale)	1b								
(Negative binomial)	1b								

Footnote: CHIT= Cambridge-Chicago Trait Compulsivity Scale; BIS= Barratt Impulsiveness Scale; SPQ=Schizotypal Personality Questionnaire; DASS-21= Depression Anxiety Stress Scale-21; DOCS=Dimensional Obsessive-Compulsive Scale

**Table 3:** Negative binomial model with intra-COVID-19 pandemic scores on the Hoarding Rating Scale

Parameter Estimates									
Parameter	B	Std. Error	95% Wald Confidence Interval		Hypothesis Test			Collinearity statistics	
			Lower	Upper	Wald Chi-Square	df	Sig.	Tolerance	VIF
(Intercept)	-3.427	1.1546	-5.690	-1.164	8.811	1	.003		
Age	.001	.0036	-.006	.008	.143	1	.705	.852	1.173
Male (vs. other) gender	.027	.0860	-.142	.195	.097	1	.756	.960	1.041
Lower (vs. higher) education levels	.162	.1481	-.128	.452	1.200	1	.273	.950	1.052
Non-white (vs. white) ethnicity	.143	.0967	-.047	.332	2.184	1	.139	.939	1.065
Non-married (vs. married) status	-.060	.0906	-.238	.117	.441	1	.507	.900	1.111
Unemployed (vs. employed)	-.265	.1794	-.617	.086	2.185	1	.139	.968	1.033
**Lack vs. presence of past HD diagnosis**	**2.708**	**1.0525**	**.645**	**4.771**	**6.620**	**1**	**.010**	.955	1.047
Negative (vs. positive) family history of HD	.103	.2765	-.439	.645	.138	1	.710	.968	1.033
Number of COVID-19 related events	.009	.0344	-.058	.076	.069	1	.792	.491	2.036
Number of COVID-19 related *stressful* events	.011	.0251	-.038	.060	.201	1	.654	.499	2.004
**CHIT total**	**.019**	**.0074**	**.004**	**.033**	**6.494**	**1**	**.011**	.719	1.391
BIS total	.011	.0097	-.008	.030	1.263	1	.261	.822	1.217
**SPQ total**	**.023**	**.0099**	**.004**	**.042**	**5.486**	**1**	**.019**	.627	1.596
DASS21 depression (before)	.017	.0138	-.010	.044	1.600	1	.206	.388	2.576
DASS21_anxiety (before)	-.009	.0206	-.049	.032	.186	1	.666	.442	2.260
DASS21_stress_(before)	-.027	.0180	-.063	.008	2.310	1	.129	.329	3.040
**HRS_total_(before)**	**.160**	**.0086**	**.143**	**.177**	**342.240**	**1**	**<.001**	.722	1.386
(Scale)	1								
(Negative binomial)	1								

Footnote: CHIT= Cambridge-Chicago Trait Compulsivity Scale; BIS= Barratt Impulsiveness Scale; SPQ=Schizotypal Personality Questionnaire; DASS-21= Depression Anxiety Stress Scale-21; HRS=Hoarding Rating Scale.

**Table 4:** Negative binomial model with intra-COVID-19 pandemic scores on the Skin Picking Scale (n=221)

Parameter Estimates									
Parameter	B	Std. Error	95% Wald Confidence Interval		Hypothesis Test			Collinearity Statistics	
			Lower	Upper	Wald Chi-Square	df	Sig.	Tolerance	VIF
(Intercept)	.893	.9224	-.914	2.701	.938	1	.333		
Age	-.002	.0072	-.016	.012	.053	1	.818	.785	1.274
Male (vs. other) gender	-.171	.1556	-.476	.134	1.206	1	.272	.912	1.097
Lower (vs. higher) education levels	-.027	.2594	-.535	.481	.011	1	.917	.935	1.069
Non-white (vs. white) ethnicity	-.109	.1814	-.464	.247	.361	1	.548	.826	1.210
Non-married (vs. married) status	.095	.1645	-.227	.418	.336	1	.562	.815	1.227
Unemployed (vs. employed)	-.125	.3268	-.765	.515	.146	1	.702	.866	1.155
Lack vs. presence of past SPD diagnosis	-.112	.4191	-.934	.709	.072	1	.789	.824	1.214
Negative (vs. positive) family history of SPD	-.016	.5679	-1.129	1.097	.001	1	.978	.717	1.394
Number of COVID-19 related events	.015	.0512	-.085	.116	.091	1	.763	.431	2.318
Number of COVID-19 related *stressful* events	.041	.0436	-.044	.127	.895	1	.344	.400	2.497
CHIT total	-.001	.0138	-.028	.026	.010	1	.921	.717	1.395
BIS total	.024	.0169	-.009	.057	2.039	1	.153	.750	1.334
SPQ total	.005	.0163	-.027	.036	.082	1	.775	.703	1.423
DASS21 depression (before)	.002	.0245	-.046	.050	.008	1	.928	.362	2.761
DASS21_anxiety (before)	-.002	.0344	-.070	.065	.005	1	.942	.339	2.946
DASS21_stress_(before)	-.012	.0317	-.074	.051	.133	1	.715	.315	3.170
**SPS_total_(before)**	**.065**	**.0170**	**.032**	**.098**	**14.765**	**1**	**<.001**	.718	1.393
(Scale)	1								
(Negative binomial)	1								

Footnote: CHIT= Cambridge-Chicago Trait Compulsivity Scale; BIS= Barratt Impulsiveness Scale; SPQ=Schizotypal Personality Questionnaire; DASS-21= Depression Anxiety Stress Scale-21; MGH-HPS= Massachusetts General Hospital Hair Pulling Scale

**Table 5:** Negative binomial model with intra-COVID-19 pandemic scores on the Dimensional Obsessive-Compulsive Scale (DOCS) as the dependent variable and pre-covid DOCS subscores as independent variables

Parameter Estimates									
Parameter	B	Std. Error	95% Wald Confidence Interval		Hypothesis Test			Collinearity Statistics	
			Lower	Upper	Wald Chi-Square	df	Sig.	Tolerance	VIF
(Intercept)	2.470	.1668	2.143	2.797	219.268	1	.000		
Age	-.005	.0032	-.011	.001	2.684	1	.101	.887	1.127
**Male (vs. other) gender**	**-.153**	**.0764**	**-.303**	**-.003**	**4.022**	**1**	**.045**	.973	1.028
Lower (vs. higher) education levels	-.093	.1325	-.353	.167	.495	1	.482	.973	1.027
Non-white (vs. white) ethnicity	.112	.0874	-.059	.284	1.656	1	.198	.923	1.084
Non-married (vs. married) status	-.050	.0788	-.205	.104	.409	1	.522	.924	1.082
Unemployed (vs. employed)	.014	.1547	-.289	.318	.009	1	.926	.974	1.027
Number of COVID-19 related events	.034	.0316	-.028	.096	1.139	1	.286	.492	2.034
**Number of COVID-19 related *stressful* events**	**.064**	**.0233**	**.019**	**.110**	**7.635**	**1**	**.006**	.514	1.946
**DOCS fear of harm - before**	**.069**	**.0176**	**.034**	**.103**	**15.198**	**1**	**<.001**	.364	2.748
DOCS contamination - before	.019	.0158	-.012	.050	1.394	1	.238	.592	1.689
**DOCS symmetry - before**	**.052**	**.0175**	**.018**	**.087**	**9.003**	**1**	**.003**	.451	2.217
DOCS unacceptable thoughts - before	.022	.0149	-.007	.051	2.175	1	.140	.420	2.380
DASS21_total_before	.004	.0046	-.005	.013	.778	1	.378	.614	1.629

Footnote: DOCS=Dimensional Obsessive-Compulsive Scale; DASS-21= Depression Anxiety Stress Scale-21

**Table 6:** Negative binomial model with intra-COVID-19 pandemic scores on the Vancouver Obsessional Compulsive Inventory-Mental Contamination (VOCI-MC)

Parameter Estimates									
Parameter	B	Std. Error	95% Wald Confidence Interval		Hypothesis Test			Collinearity Statistics	
			Lower	Upper	Wald Chi-Square	df	Sig.	Tolerance	VIF
(Intercept)	.088	.4074	-.710	.887	.047	1	.829		
Age	-.004	.0034	-.010	.003	1.210	1	.271	.845	1.184
Male (vs. other) gender	-.102	.0810	-.260	.057	1.569	1	.210	.943	1.060
Lower (vs. higher) education levels	-.118	.1394	-.391	.155	.718	1	.397	.951	1.052
**Non-white (vs. white) ethnicity**	**.208**	**.0900**	**.032**	**.385**	**5.350**	**1**	**.021**	.931	1.074
Non-married (vs. married) status	.035	.0840	-.129	.200	.178	1	.673	.898	1.114
Unemployed (vs. employed)	-.207	.1625	-.526	.111	1.628	1	.202	.969	1.032
Lack vs. presence of past OCD diagnosis	.208	.2074	-.198	.615	1.008	1	.315	.870	1.149
Negative (vs. positive) family history of OCD	.053	.1630	-.266	.373	.107	1	.743	.902	1.109
Number of COVID-19 related events	.035	.0345	-.033	.102	1.005	1	.316	.494	2.024
**Number of COVID-19 related *stressful* events**	**.056**	**.0257**	**.006**	**.107**	**4.822**	**1**	**.028**	.500	1.999
**CHIT total**	**.035**	**.0070**	**.021**	**.049**	**24.677**	**1**	**<.001**	.702	1.424
BIS total	.004	.0088	-.013	.021	.229	1	.632	.819	1.221
**SPQ total**	**.031**	**.0093**	**.013**	**.049**	**11.195**	**1**	**.001**	.612	1.633
DASS21 depression (before)	.007	.0130	-.018	.033	.300	1	.584	.392	2.552
DASS21_anxiety (before)	-.025	.0212	-.067	.017	1.396	1	.237	.381	2.622
DASS21_stress_(before)	-.010	.0175	-.044	.025	.307	1	.580	.329	3.040
**VOCI-MC total (before)**	**.069**	**.0056**	**.059**	**.080**	**155.275**	**1**	**<.001**	.508	1.969
(Scale)	1								
(Negative binomial)	1								

Footnote: CHIT= Cambridge-Chicago Trait Compulsivity Scale; BIS= Barratt Impulsiveness Scale; SPQ=Schizotypal Personality Questionnaire; DASS-21= Depression Anxiety Stress Scale-21; VOCI=Vancouver Obsessional Compulsive Inventory-Mental Contamination.
