# Supplementary material for: Correlates of obsessive-compulsive and related disorders symptom severity during the COVID-19 pandemic
Source: J Psychiatr Res. Author manuscript; Available in PMC 2021 Nov 1. (PMC8548281; doi:10.1016/j.jpsychires.2021.03.046)
Supplement: Appendix [file EMS127423-supplement-Appendix.docx]

Appendix

Table 1 of appendix: Negative binomial model with intra-COVID-19 pandemic scores on the Anxiety Appearance Inventory

| Parameter Estimates | | | | | | | |  | |
| --- | --- | --- | --- | --- | --- | --- | --- | --- | --- |
| Parameter | B | Std. Error | 95% Wald Confidence Interval | | Hypothesis Test | | | Collinearity Statistics | |
|  |  |  | Lower | Upper | Wald Chi-Square | df | Sig. | Toler-ance | VIF |
| (Intercept) | -.048 | .5127 | -1.053 | .957 | .009 | 1 | .926 |  |  |
| Age | -.002 | .0035 | -.008 | .005 | .230 | 1 | .632 | .821 | 1.217 |
| Male (vs. other) gender | -.144 | .0827 | -.306 | .018 | 3.027 | 1 | .082 | .919 | 1.088 |
| Lower (vs. higher) education levels | .074 | .1417 | -.204 | .351 | .270 | 1 | .603 | .948 | 1.055 |
| Non-white (vs. white) ethnicity | .033 | .0928 | -.149 | .215 | .124 | 1 | .724 | .930 | 1.076 |
| Non-married (vs. married) status | .153 | .0850 | -.014 | .319 | 3.221 | 1 | .073 | .900 | 1.111 |
| Unemployed (vs. employed) | -.068 | .1656 | -.392 | .257 | .167 | 1 | .683 | .970 | 1.031 |
| Lack vs. presence of past BDD diagnosis | .071 | .3447 | -.605 | .747 | .042 | 1 | .837 | .779 | 1.284 |
| Negative (vs. positive) family history of BDD | .311 | .3228 | -.322 | .943 | .926 | 1 | .336 | .795 | 1.257 |
| Number of COVID-19 related events | .029 | .0298 | -.029 | .088 | .963 | 1 | .326 | .495 | 2.018 |
| Number of COVID-19 related *stressful* events | .025 | .0227 | -.019 | .070 | 1.224 | 1 | .269 | .499 | 2.004 |
| **CHIT total** | **.015** | **.0071** | **.001** | **.028** | **4.171** | **1** | **.041** | .701 | 1.427 |
| BIS total | .005 | .0092 | -.013 | .023 | .258 | 1 | .612 | .807 | 1.240 |
| SPQ total | .014 | .0093 | -.004 | .033 | 2.355 | 1 | .125 | .623 | 1.606 |
| DASS21 depression (before) | -.002 | .0141 | -.029 | .026 | .016 | 1 | .898 | .370 | 2.704 |
| DASS21_anxiety (before) | .013 | .0203 | -.027 | .053 | .396 | 1 | .529 | .428 | 2.334 |
| DASS21_stress_(before) | -.002 | .0177 | -.037 | .033 | .013 | 1 | .908 | .323 | 3.094 |
| **AAI_total_(before)** | **.093** | **.0074** | **.079** | **.108** | **159.87** | **1** | **<.001** | .567 | 1.764 |
| (Scale) | 1 |  |  |  |  |  |  |  |  |
| (Negative binomial) | 1 |  |  |  |  |  |  |  |  |

Footnote: CHIT= Cambridge-Chicago Trait Compulsivity Scale; BIS= Barratt Impulsiveness Scale; SPQ=Schizotypal Personality Questionnaire; DASS-21= Depression Anxiety Stress Scale-21; AAI=Anxiety Appearance Inventory

Table 2 of appendix: Negative binomial model with intra-COVID-19 pandemic scores on the Massachusetts General Hospital Hair Pulling Scale (n=98)

| Parameter Estimates | | | | | | | |  | |  |
| --- | --- | --- | --- | --- | --- | --- | --- | --- | --- | --- |
| Parameter | B | Std. Error | 95% Wald Confidence Interval | | Hypothesis Test | | | Collinearity statistics | | |
|  |  |  | Lower | Upper | Wald Chi-Square | df | Sig. | Tolerance | VIF | |
| (Intercept) | 1.760 | 1.2286 | -.648 | 4.168 | 2.052 | 1 | .152 |  |  | |
| Age | -.004 | .0125 | -.029 | .020 | .126 | 1 | .723 | .743 | 1.346 | |
| Male (vs. other) gender | -.201 | .2866 | -.763 | .361 | .491 | 1 | .484 | .642 | 1.557 | |
| Lower (vs. higher) education levels | -.183 | .4023 | -.972 | .605 | .207 | 1 | .649 | .856 | 1.168 | |
| Non-white (vs. white) ethnicity | -.200 | .2665 | -.723 | .322 | .565 | 1 | .452 | .834 | 1.199 | |
| Non-married (vs. married) status | .170 | .2854 | -.389 | .729 | .355 | 1 | .551 | .714 | 1.400 | |
| Unemployed (vs. employed) | .171 | .6595 | -1.122 | 1.464 | .067 | 1 | .795 | .706 | 1.416 | |
| Lack vs. presence of past TTM diagnosis | .435 | .5342 | -.612 | 1.482 | .663 | 1 | .415 | .521 | 1.919 | |
| Negative (vs. positive) family history of TTM | -.066 | .5596 | -1.163 | 1.031 | .014 | 1 | .906 | .716 | 1.397 | |
| Number of COVID-19 related events | .024 | .0797 | -.132 | .180 | .090 | 1 | .765 | .254 | 3.933 | |
| Number of COVID-19 related *stressful* events | .006 | .0725 | -.136 | .148 | .006 | 1 | .938 | .272 | 3.674 | |
| CHIT total | .006 | .0210 | -.035 | .047 | .087 | 1 | .767 | .666 | 1.502 | |
| BIS total | -.014 | .0297 | -.072 | .044 | .220 | 1 | .639 | .604 | 1.655 | |
| SPQ total | .002 | .0260 | -.049 | .052 | .004 | 1 | .951 | .670 | 1.491 | |
| DASS21 depression (before) | -.041 | .0416 | -.123 | .041 | .966 | 1 | .326 | .316 | 3.169 | |
| DASS21_anxiety (before) | .051 | .0499 | -.047 | .149 | 1.057 | 1 | .304 | .309 | 3.233 | |
| DASS21_stress_(before) | .000 | .0465 | -.091 | .091 | .000 | 1 | .996 | .340 | 2.943 | |
| **MGHHPS_total_(before)** | **.071** | **.0287** | **.014** | **.127** | **6.065** | **1** | **.014** | .550 | 1.820 | |
| (Scale) | 1 |  |  |  |  |  |  |  |  | |
| (Negative binomial) | 1 |  |  |  |  |  |  |  |  | |

Footnote: CHIT= Cambridge-Chicago Trait Compulsivity Scale; BIS= Barratt Impulsiveness Scale; SPQ=Schizotypal Personality Questionnaire; DASS-21= Depression Anxiety Stress Scale-21; MGH-HPS= Massachusetts General Hospital Hair Pulling Scale
